# Lived experiences, barriers, and facilitators of voluntary non-suicidal self-injury disclosure among adolescents who have disclosed: a qualitative systematic review protocol

**DOI:** 10.3389/fpubh.2026.1964572

**Published:** 2026-10-27

**Authors:** Xubin He, Xinghong Xu, Hongyan Huang, Jing Peng, Bo Yang, Jingtong Huang

**Affiliations:** Chongqing Mental Health Center, Chongqing, China

**Keywords:** adolescent, disclosure, lived experience, meta-aggregation, non-suicidal self-injury, qualitative research

## Abstract

**Background:**

Non-suicidal self-injury (NSSI) is common among adolescents and often remains concealed because of shame and stigma. Voluntary disclosure can provide access to support, but emotional and interpersonal concerns shape the decision to disclose. This review will synthesize disclosure experiences, barriers, and facilitators reported by adolescents who have voluntarily disclosed NSSI at least once.

**Methods:**

Primary qualitative studies and separately extractable qualitative components of mixed-methods studies involving adolescents aged 10–19 years will be eligible. Searches will cover PubMed, Embase, APA PsycINFO, CINAHL, Scopus, and Web of Science Core Collection. China National Knowledge Infrastructure, Wanfang Data, and the Chinese Biomedical Literature Database will also be searched. Coverage will extend from inception through 31 March 2026, inclusive, without language restrictions; formal searches have not yet been executed. Gray literature, reference lists, and forward citations will also be searched. Two human reviewers will independently select studies, appraise them using the Joanna Briggs Institute (JBI) qualitative checklist, and extract findings. Findings will be synthesized using JBI meta-aggregation, with confidence assessed using ConQual.

**Discussion:**

The synthesis will examine disclosure decisions, recipients' responses, and subsequent experiences from adolescents' perspectives. Findings may inform clinical, school, and family support for voluntary NSSI disclosure.

**Systematic review registration:**

https://www.crd.york.ac.uk/PROSPERO/view/CRD420261355173, identifier: CRD420261355173.

## 1 Introduction

Non-suicidal self-injury (NSSI) refers to the intentional and direct damage of one's own body tissue without suicidal intent and for purposes that are not socially sanctioned (1). Common forms include cutting, hitting, and burning. NSSI may function as a maladaptive strategy for regulating negative emotions, alleviating psychological distress, or punishing oneself (2). Reflecting its distinct clinical profile, the Diagnostic and Statistical Manual of Mental Disorders, Fifth Edition (DSM-5), lists NSSI as a condition for further study, separate from suicidal behavior and broader self-harm constructs (1).

Adolescents are at particularly high risk for NSSI, with a global lifetime prevalence of approximately 17.7% (3). NSSI commonly begins around the age of 12, peaks during middle adolescence between the ages of 14 and 16, and may decline after the age of 18 (4). Its adverse effects are not limited to physical injury. NSSI commonly co-occurs with depression and substance use problems and is associated with subsequent suicidal thoughts and behaviors (5, 6). In a longitudinal meta-analysis, a history of NSSI was associated with a weighted mean odds ratio of 4.27 for a subsequent suicide attempt (7). Despite its clinical significance, NSSI is frequently concealed because of secrecy, shame, and social stigma (8, 9). Voluntary disclosure may provide an important opportunity for adolescents to communicate their distress and access interpersonal or professional support (10, 11). Understanding how adolescents disclose their NSSI is therefore essential for developing timely and responsive support and intervention strategies.

Positive disclosure experiences may promote emotional relief, social connectedness, access to support, and motivation to reduce NSSI (11, 12). Disclosure may also reduce secrecy and challenge internalized NSSI-related stigma (13). However, fewer than half of adolescents who engage in NSSI disclose their behavior, and an even smaller proportion seek professional support (14). This contrast between the potential benefits of disclosure and the relatively low disclosure rate indicates that disclosure is a complex and highly individualized process.

The Disclosure Processes Model provides a useful theoretical framework for understanding this process (15). It conceptualizes disclosure as a dynamic process shaped by antecedent goals, the disclosure event, and post-disclosure outcomes. In the context of NSSI, adolescents may balance approach-oriented goals, such as obtaining support, emotional relief, and understanding, against avoidance-oriented goals, such as avoiding stigma, burdening others, or losing control over treatment decisions. Recipients' responses and adolescents' post-disclosure experiences may subsequently influence whether, when, how, and to whom they disclose in the future (15). However, the model provides a general account of disclosing concealable stigmatized information and was not developed specifically for adolescent NSSI. It may therefore not fully capture how developmental needs, confidentiality concerns, perceived loss of autonomy, disclosure recipients, and sociocultural contexts shape adolescents' actual disclosure experiences.

Although quantitative studies have examined associations between disclosure and factors such as NSSI frequency, severity, and demographic characteristics, these variables alone may not adequately capture the emotional trade-offs, interpersonal dynamics, and contextual considerations involved in disclosure to peers, parents, or healthcare professionals (14, 16). An in-depth synthesis of adolescents' lived experiences of voluntary NSSI disclosure, together with their perceived barriers and facilitators, is therefore needed to inform more responsive identification and support systems.

Qualitative studies have explored adolescents' experiences of NSSI disclosure, but the evidence remains fragmented across sociocultural contexts, disclosure recipients, and service settings. A preliminary search of PubMed, the Cochrane Database of Systematic Reviews, and JBI Evidence Synthesis, conducted in March 2026, identified three relevant reviews. Simone and Hamza (14) summarized NSSI disclosure rates, recipients, barriers and facilitators, functions, and post-disclosure outcomes across adolescent and young adult populations. However, their review integrated heterogeneous evidence narratively and did not specifically synthesize adolescents' first-person qualitative accounts of voluntary disclosure. More recently, Hon et al. (17) applied Social Cognitive Theory in a scoping review to map the social-cognitive factors underlying NSSI disclosure, particularly disclosure self-efficacy, outcome expectancies, and social modeling. Although theoretically informative, their review was not designed to critically appraise and meta-aggregate adolescents' lived experiences of voluntary NSSI disclosure or assess confidence in the synthesized qualitative findings. Lewis and Seko (12) synthesized evidence on the benefits and risks of online NSSI activities, including emotional self-disclosure, peer interaction, exposure to NSSI-related content, and participation in online communities. However, their broad focus on online activities did not specifically examine adolescents' voluntary disclosure experiences across both digital and offline settings.

This review will use the Joanna Briggs Institute (JBI) meta-aggregation to appraise and synthesize qualitative evidence on voluntary NSSI disclosure. The question follows the Population, Phenomenon of Interest, and Context (PICo) structure. How do adolescents aged 10–19 years who have voluntarily disclosed NSSI describe their disclosure experiences, barriers, and facilitators across interpersonal, service, and digital contexts?

## 2 Methods and analysis

The review will follow JBI guidance for qualitative evidence synthesis (18). This protocol follows the Preferred Reporting Items for Systematic Review and Meta-Analysis Protocols (PRISMA-P) statement (19). The completed review will follow the Preferred Reporting Items for Systematic Reviews and Meta-Analyses (PRISMA) 2020 statement (20). The review was prospectively registered in PROSPERO, the International Prospective Register of Systematic Reviews (CRD420261355173).

### 2.1 Eligibility criteria

#### 2.1.1 Participants

Eligible participants are adolescents aged 10–19 years, consistent with the World Health Organization definition (21), who have voluntarily disclosed their own NSSI at least once. Mixed-age studies must provide findings and supporting illustrations attributable to this age group. Adolescents who have never voluntarily disclosed NSSI will not be directly represented.

#### 2.1.2 Phenomenon of interest

Voluntary disclosure is an adolescent's intentional communication about their own NSSI to a person, group, or online audience, without coercion or compelled acknowledgment. Communication may be spontaneous or prompted, direct or indirect, verbal or nonverbal, and may occur face-to-face, digitally, or anonymously. Voluntariness concerns the adolescent's choice to communicate, regardless of who initiated the interaction. The review will examine disclosure decisions, events, and subsequent experiences, including barriers and facilitators reported by adolescents who have disclosed.

Prompted, observation-triggered, indirect, nonverbal, digital, anonymous, and research-interview disclosures are eligible when communicative intent is clear. Research-context episodes must be examined as disclosure experiences and will be coded separately. Accidental observation, third-party reporting, and compelled acknowledgment are excluded. Intentional disclosures following involuntary discovery are eligible only when separately identifiable. Mixed reports must provide separately attributable voluntary NSSI findings and supporting illustrations. Supplementary Appendix V provides the operational taxonomy, decision tree, and examples.

For studies using self-harm or related umbrella terms, definitions, assessments, explicit accounts, or author clarification must establish that the disclosed behavior meets the NSSI definition. Findings and supporting illustrations must be separately attributable to NSSI disclosure. Eligibility concerns the disclosed behavior; a history of suicidal thoughts or attempts does not itself exclude a participant. Findings involving suicidal, mixed, or unresolved intent will be excluded (Supplementary Appendix V).

#### 2.1.3 Context

Studies from any geographic or sociocultural setting will be eligible, including family, peer, educational, healthcare, community, and digital settings. There will be no restrictions on formal or informal disclosure recipients.

#### 2.1.4 Types of studies

Eligible evidence comprises primary qualitative studies and separately extractable qualitative components of mixed-methods research. Designs include phenomenology, grounded theory, ethnography, action research, narrative inquiry, qualitative case studies, and descriptive qualitative research. Mixed-methods reports must describe their qualitative methods and distinguish qualitative findings from quantitative results; uncertain separability will prompt author clarification. Open-ended survey responses qualify only when analyzed using a reported qualitative method and presented as extractable findings. Quantitative-only studies, secondary syntheses, clinical case reports without systematic qualitative analysis, theoretical or opinion papers, editorials, and policy documents will be excluded.

#### 2.1.5 Review outcomes

The primary outcome comprises synthesized qualitative findings on three domains of voluntary NSSI disclosure among adolescents who have disclosed at least once. These are experiences before, during, and after disclosure; barriers that hindered or delayed disclosure; and conditions or motives that facilitated it. No additional outcomes or numerical effect measures are prespecified, and these domains will not determine synthesis categories.

### 2.2 Search strategy

A preliminary PubMed search on 31 March 2026 informed term development; formal comprehensive searches have not yet been executed. Supplementary Appendix I gives the planned strategies, interfaces, fields, controlled vocabulary, limits, and source records. The primary route combines self-injury, adolescent population, and disclosure terms; a complementary route combines self-injury, population, and qualitative terms. Their union covers broader self-harm terminology, telling, revealing, concealment, and help-seeking without excluding suicide-related records at retrieval. The search strategy has not undergone peer review using the Peer Review of Electronic Search Strategies (PRESS) framework (22). Search methods and results will be reported in accordance with the PRISMA extension for searches (PRISMA-S) (23).

Searches will cover PubMed, Embase (Ovid), APA PsycINFO (Ovid), CINAHL (EBSCOhost), Scopus, and Web of Science Core Collection. Chinese sources comprise China National Knowledge Infrastructure (CNKI), Wanfang Data, and the Chinese Biomedical Literature Database (CBM). Strategies will use source-specific syntax and controlled vocabulary. Reference lists of included studies and relevant reviews will be checked, alongside forward citation searches. ProQuest Dissertations and Theses Global and relevant university or organizational repositories will be searched for gray literature and unpublished reports. Authors and field experts may be contacted to identify report citations or access information.

Publication coverage will extend from database inception through 31 March 2026, inclusive, without language restrictions. Actual execution dates and retrieval counts will be recorded separately for each source. When an interface cannot apply a day-level cutoff, publication dates will be checked during screening. Searches will be rerun before synthesis to capture newly indexed records within this coverage window. Machine translation may help reviewers read records; it will not generate eligibility recommendations. Full texts, culturally specific expressions, and participant quotations will be checked with professional language support as needed. Consequential translation decisions and residual uncertainty will be documented.

### 2.3 Study selection

Records will be imported into EndNote 20 (Clarivate, London, United Kingdom). Exact identifiers and bibliographic fields will identify duplicate candidates, which reviewers will verify before removal. Source tags and a duplicate log will be retained. Multiple reports from one study will be linked and treated as a single study; all relevant reports will inform extraction. Unique records will be transferred to JBI SUMARI, the System for the Unified Management, Assessment and Review of Information (24). Supplementary Appendix I specifies the deduplication procedure and reporting fields.

Two reviewers will independently pilot screening on a random 10% of deduplicated records, rounded up and bounded by 100 and 200 records. If fewer than 100 records are available, all will be used. Reviewers will lock decisions before comparison and report the decision table, agreement, disagreements, and Gwet's AC1 with a 95% confidence interval (25). Formal screening requires raw agreement of at least 80%, AC1 of at least 0.80, and clarification of all disagreements. Failed rounds will prompt clarification of the screening instructions and repeat calibration. Supplementary Appendix VI specifies the rationale, repeat procedures, and third-reviewer training after three consecutive failed rounds.

Two human reviewers will independently screen all titles and abstracts and assess potentially eligible or uncertain records at full text. They will apply the intent, disclosure, and separability rules in Supplementary Appendix V. Machine learning will not prioritize records or recommend eligibility, and inclusion or exclusion decisions will not be automated. Software and translation support will not replace either reviewer's judgment.

Screening disagreements will be resolved through discussion or adjudication by a third reviewer. Authors will be emailed for essential eligibility information, with a reminder on day 14 and a decision on day 28 using available evidence. Findings with unresolved intent or separability will be excluded; studies with no eligible findings will be excluded at full text. Appendix V specifies the contact procedure and decision records.

Full-text exclusions will be documented with reasons, and the completed review will include a populated PRISMA 2020 flow diagram (20). The planned flow-diagram template is shown in Figure 1.

**Figure 1:**
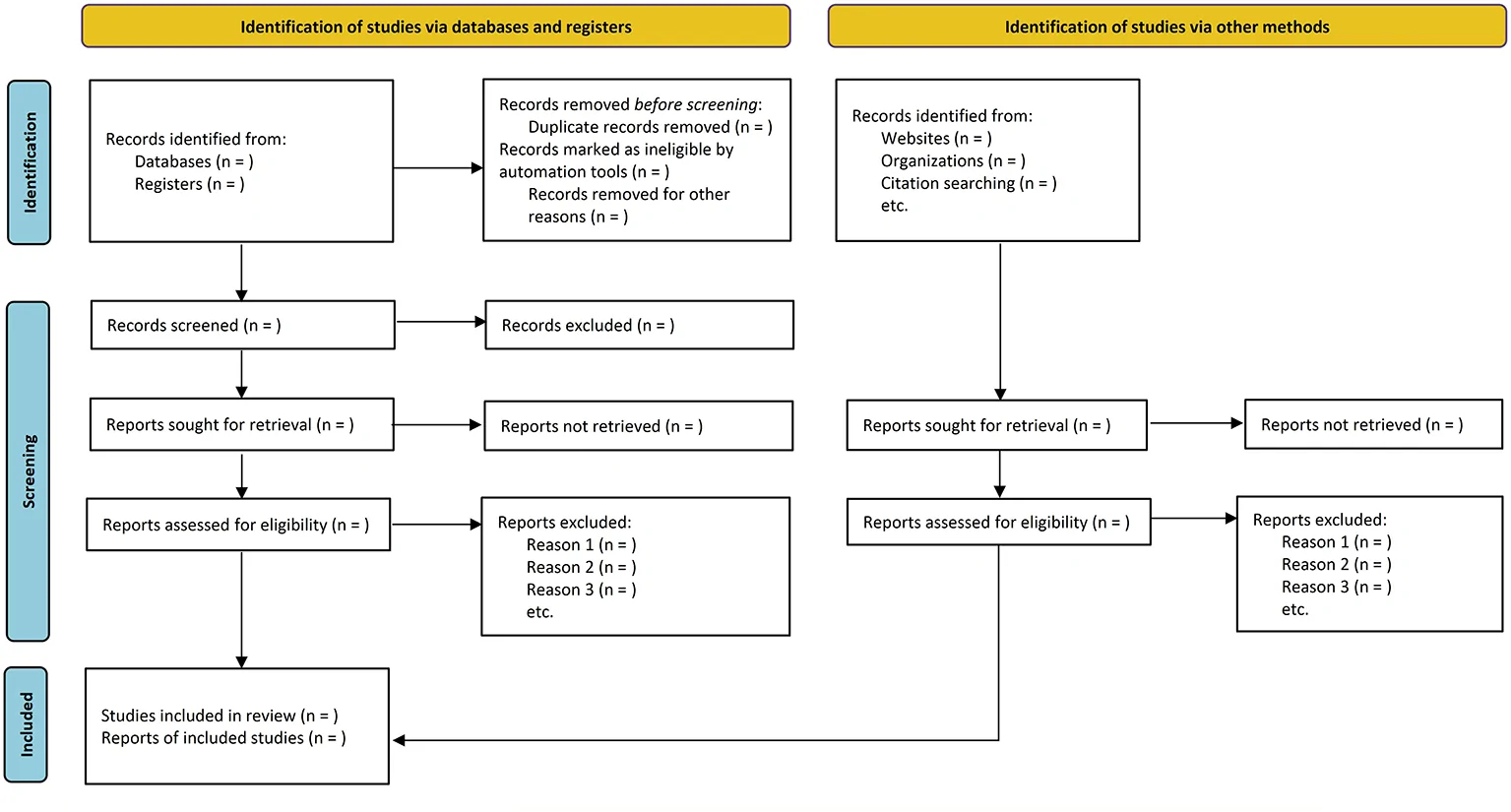
PRISMA 2020 flow diagram for planned study selection (20).

### 2.4 Assessment of methodological quality

Two human reviewers will independently appraise each study using the JBI Critical Appraisal Checklist for Qualitative Research (Supplementary Appendix II) (18). It assesses methodological congruity, representation of participants' voices, researcher reflexivity, ethics, and whether conclusions follow from the analysis.

Before formal appraisal, reviewers will independently assess two randomly selected studies and resolve differences in their interpretation of the criteria.

Items will be rated Yes, No, Unclear, or Not applicable. No study will be excluded solely for low methodological quality; appraisal will inform interpretation and the ConQual assessment.

Appraisal disagreements will be discussed, with unresolved judgments adjudicated by a third reviewer. Missing methodological details will be sought through the contact procedure in Supplementary Appendix V; unresolved items will remain Unclear. Appraisal results will be reported in narrative and tabular formats.

### 2.5 Data extraction

Two human reviewers will independently extract data using the JBI SUMARI qualitative extraction framework (24), piloted on five studies. Supplementary Appendix III specifies the study characteristics and extraction fields. Each author-reported primary finding and supporting illustration will be retained verbatim, linked to its source and context, and extracted without DPM labels. Extraction will document NSSI intent, separability, disclosure pathway, recipient, communication mode, and preceding involuntary discovery.

Evidence will come from study reports and publicly available supplements. Data queries to authors will clarify definitions, methods, attribution, separability, or reported supporting material, following the schedule in Supplementary Appendix V. Raw interviews, complete primary-study datasets, and individual participant data (IPD) will not be requested or obtained. Author clarifications will be linked to reported evidence and will not be reanalyzed as new primary findings.

After eligibility is established, reviewers will rate findings as Unequivocal, Credible, or Unsupported according to their congruence with supporting illustrations. Unresolved methodological or supporting-material gaps will be judged from the available report. Disagreements will be resolved through discussion or a third reviewer.

### 2.6 Data synthesis

Only Unequivocal and Credible findings will enter meta-aggregation. Unsupported findings will be logged separately and excluded before categorization; they will not contribute to ConQual. Eligible findings that cannot be combined without changing their meaning will be presented separately. If meta-aggregation is infeasible, a structured narrative summary will explain why aggregation was not possible.

Two reviewers will independently group eligible findings by similarity of meaning and aggregate categories into synthesized findings using JBI SUMARI (26), preserving context. Differences will be discussed; a third reviewer will verify source links and adjudicate unresolved disagreements. After synthesis and ConQual assessments are finalized, synthesized findings will be mapped to DPM concepts for interpretation (15). Mapping will not alter source wording, categories, synthesized findings, or confidence ratings. Divergent and unmapped findings will be retained, following the sequence and audit trail in Supplementary Appendix VI.

### 2.7 Reflexivity

Reflexivity will be considered throughout data extraction, categorization, and synthesis. The review team will discuss how disciplinary backgrounds, clinical experience, and prior assumptions may influence interpretation. Because JBI meta-aggregation seeks to preserve the meanings of primary study findings, extensive reinterpretation will be minimized. Key analytic decisions will be documented through reflexive discussions and an audit trail to enhance transparency.

### 2.8 Assessing confidence in synthesized findings

ConQual assessment will begin at High confidence for each synthesized finding (27). Dependability and credibility downgrades will be added, with a minimum final rating of very low. Supplementary Appendix IV gives the decision rules and Summary of Findings fields.

Dependability will use JBI items Q2, Q3, Q4, Q6, and Q7. Four or five “Yes” ratings entail no downgrade, two or three entail one level, and zero or one entail two levels. Each primary finding inherits its source-study rating. The rating shared by more than half of contributing findings determines the synthesized finding's dependability; without a majority, the lowest contributing rating applies. This aggregation rule is prespecified for this review. Credibility entails no downgrade for all Unequivocal findings, one level for mixed Unequivocal and Credible findings, and two levels for all Credible findings.

Two reviewers will independently record the contributing evidence, domain judgments, and final ConQual ratings before comparison. Unresolved disagreements will be independently adjudicated by a third reviewer. The Summary of Findings table will report final ratings and reasons for downgrading.

### 2.9 Patient and public involvement

Adolescents with NSSI disclosure experience, family members, carers, and service-user representatives were not involved in developing this protocol. No direct involvement is currently planned in selection, appraisal, extraction, synthesis, interpretation, or dissemination. A youth-advisory process and the necessary support and safeguarding arrangements have not been established.

## 3 Discussion

This review will examine how adolescents who have disclosed NSSI weigh its benefits and risks, experience recipients' responses, and describe subsequent help-seeking. Bringing these accounts together may help clinicians, school personnel, families, and policymakers identify conditions that support voluntary disclosure. The synthesis may also identify questions for future qualitative and intervention research.

Several limitations are anticipated. Findings that combine suicidal and non-suicidal self-injury without clear separability, or for which intent remains unresolved, will be excluded, limiting the evidence available for synthesis. Because eligibility requires at least one voluntary disclosure, the synthesis will not directly capture barriers, expectations, or conditions for disclosure reported by adolescents with NSSI who have never voluntarily disclosed. Differences in disclosure recipients, settings, and sociocultural contexts may limit comparability across studies. Translation may also reduce culturally specific meanings and nuances. The absence of direct involvement by adolescents with lived experience of NSSI may limit how well the review question and interpretation reflect their priorities and perspectives. The synthesis will also depend on the richness of the reported findings and the availability of supporting participant quotations or illustrations.

## Supplementary material

Supplementary Appendices I-VI: Search strategies, appraisal and extraction forms, ConQual rules, eligibility criteria, and review procedures.
