# Supplementary material for: Lived experiences, barriers, and facilitators of voluntary non-suicidal self-injury disclosure among adolescents who have disclosed: a qualitative systematic review protocol
Source: Front Public Health. 2026 Oct 27;14:1964572. doi: 10.3389/fpubh.2026.1964572 (PMC13748222; doi:10.3389/fpubh.2026.1964572)
Supplement: Supplementary file 1 [file Supplementary_file_1.docx]

**Appendix I: Search strategies and search records**

| **Preliminary PubMed search used for strategy development** Interface: NLM PubMed \| Search conducted: 31 March 2026 | | |
| --- | --- | --- |
| **Search** | **Query** | **Records retrieved** |
| #1 | "Self-Injurious Behavior"[MeSH Terms] OR "nonsuicidal self-injury"[Title/Abstract] OR "non-suicidal self-injury"[Title/Abstract] OR "nonsuicidal self injury"[Title/Abstract] OR NSSI[Title/Abstract] OR self-injur*[Title/Abstract] OR self-harm*[Title/Abstract] OR self-cut*[Title/Abstract] OR self-mutilat*[Title/Abstract] OR "deliberate self-harm"[Title/Abstract] OR DSH[Title/Abstract] | 104,813 |
| #2 | "Adolescent"[MeSH Terms] OR "Child"[MeSH Terms] OR adolescen*[Title/Abstract] OR teen*[Title/Abstract] OR youth*[Title/Abstract] OR "young person"[Title/Abstract] OR "young people"[Title/Abstract] OR "young adult"[Title/Abstract] OR "young adults"[Title/Abstract] | 3,890,527 |
| #3 | "Self Disclosure"[MeSH Terms] OR disclos*[Title/Abstract] OR "opening up"[Title/Abstract] OR "telling others"[Title/Abstract] OR reveal*[Title/Abstract] | 3,556,560 |
| #4 | "Qualitative Research"[MeSH Terms] OR "Focus Groups"[MeSH Terms] OR "Interviews as Topic"[MeSH Terms] OR qualitative[Title/Abstract] OR interview*[Title/Abstract] OR "focus group"[Title/Abstract] OR "focus groups"[Title/Abstract] OR phenomenolog*[Title/Abstract] OR ethnograph*[Title/Abstract] OR "grounded theory"[Title/Abstract] OR "thematic analysis"[Title/Abstract] OR "content analysis"[Title/Abstract] OR narrative*[Title/Abstract] OR "mixed methods"[Title/Abstract] OR "mixed-methods"[Title/Abstract] | 1,057,946 |
| #5 | #1 AND #2 AND #3 AND #4 | 453 |
| Purpose | Limited preliminary search for term development only; not the formal comprehensive search and not included in review source totals. | 438 reported |

| **PubMed** Interface: NLM PubMed \| Formal search execution date: not yet executed | | |
| --- | --- | --- |
| **Search** | **Query** | **Records retrieved** |
| #1 | "Self-Injurious Behavior"[Mesh] OR "nonsuicidal self-injury"[tiab] OR "non-suicidal self-injury"[tiab] OR "nonsuicidal self injury"[tiab] OR "non suicidal self injury"[tiab] OR NSSI[tiab] OR "self injur*"[tiab] OR "self-injur*"[tiab] OR "self harm*"[tiab] OR "self-harm*"[tiab] OR selfinjur*[tiab] OR selfharm*[tiab] OR "self mutilat*"[tiab] OR "self-mutilat*"[tiab] OR "self cut*"[tiab] OR "self-cut*"[tiab] OR "deliberate self harm"[tiab] OR DSH[tiab] | Not yet executed |
| #2 | "Adolescent"[Mesh] OR "Child"[Mesh] OR "Young Adult"[Mesh] OR adolescen*[tiab] OR teen*[tiab] OR youth*[tiab] OR juvenile*[tiab] OR "young person*"[tiab] OR "young people"[tiab] OR "young adult*"[tiab] OR schoolchild*[tiab] OR student*[tiab] | Not yet executed |
| #3 | "Self Disclosure"[Mesh] OR "Help-Seeking Behavior"[Mesh] OR disclos*[tiab] OR nondisclos*[tiab] OR "non-disclos*"[tiab] OR tell[tiab] OR tells[tiab] OR telling[tiab] OR told[tiab] OR reveal*[tiab] OR conceal*[tiab] OR hide[tiab] OR hides[tiab] OR hiding[tiab] OR hidden[tiab] OR secret*[tiab] OR secrecy[tiab] OR confid*[tiab] OR admit*[tiab] OR communicat*[tiab] OR "opening up"[tiab] OR "open up"[tiab] OR "help seek*"[tiab] OR "help-seek*"[tiab] OR helpseek*[tiab] OR "seek* help"[tiab] OR "support seek*"[tiab] OR "support-seek*"[tiab] | Not yet executed |
| #4 | "Qualitative Research"[Mesh] OR "Interviews as Topic"[Mesh] OR "Focus Groups"[Mesh] OR qualitative[tiab] OR interview*[tiab] OR "focus group*"[tiab] OR phenomenolog*[tiab] OR ethnograph*[tiab] OR "grounded theory"[tiab] OR "thematic analys*"[tiab] OR "content analys*"[tiab] OR "framework analys*"[tiab] OR "interpretative phenomenological analys*"[tiab] OR narrative*[tiab] OR "lived experience*"[tiab] OR experience*[tiab] OR perception*[tiab] OR perspective*[tiab] OR "mixed method*"[tiab] OR "mixed-method*"[tiab] OR "open ended"[tiab] OR "open-ended"[tiab] OR "action research"[tiab] OR "case stud*"[tiab] | Not yet executed |
| #5 | #1 AND #2 AND #3 | Not yet executed |
| #6 | #1 AND #2 AND #4 | Not yet executed |
| #7 | #5 OR #6 | Not yet executed |
| Limits | Apply the publication cutoff of 31 March 2026, with no lower date bound; inspect and archive Search Details and warnings before export. | Not yet available |

| **Embase** Interface: Ovid \| Formal search execution date: not yet executed | | |
| --- | --- | --- |
| **Search** | **Query** | **Records retrieved** |
| 1 | exp self injury/ or ((self adj1 (injur* or harm* or mutilat* or cut*)) or selfinjur* or selfharm* or NSSI or DSH or (nonsuicidal adj2 injur*) or (non suicidal adj2 injur*)).ti,ab. | Not yet executed |
| 2 | exp adolescent/ or exp child/ or young adult/ or (adolescen* or teen* or youth* or juvenile* or young person* or young people or young adult* or schoolchild* or student*).ti,ab. | Not yet executed |
| 3 | self disclosure/ or help seeking behavior/ or (disclos* or nondisclos* or (non adj1 disclos*) or tell or tells or telling or told or reveal* or conceal* or hide or hides or hiding or hidden or secret* or secrecy or confid* or admit* or communicat* or (open* adj1 up) or ((help or support) adj3 seek*) or helpseek*).ti,ab. | Not yet executed |
| 4 | qualitative research/ or interview/ or focus group/ or (qualitative or interview* or (focus adj1 group*) or phenomenolog* or ethnograph* or (grounded adj1 theory) or ((thematic or content or framework) adj1 analys*) or (interpretative adj1 phenomenological adj1 analys*) or narrative* or (lived adj1 experience*) or experience* or perception* or perspective* or (mixed adj1 method*) or (open adj1 ended) or (action adj1 research) or (case adj1 stud*)).ti,ab. | Not yet executed |
| 5 | 1 and 2 and 3 | Not yet executed |
| 6 | 1 and 2 and 4 | Not yet executed |
| 7 | 5 or 6 | Not yet executed |
| Limits | Publication coverage through 31 March 2026; no other limits. | Not yet available |

| **APA PsycINFO** Interface: Ovid \| Formal search execution date: not yet executed | | |
| --- | --- | --- |
| **Search** | **Query** | **Records retrieved** |
| 1 | exp Self Injurious Behavior/ or ((self adj1 (injur* or harm* or mutilat* or cut*)) or selfinjur* or selfharm* or NSSI or DSH or (nonsuicidal adj2 injur*) or (non suicidal adj2 injur*)).ti,ab. | Not yet executed |
| 2 | exp Adolescence/ or exp Childhood/ or Young Adulthood/ or (adolescen* or teen* or youth* or juvenile* or young person* or young people or young adult* or schoolchild* or student*).ti,ab. | Not yet executed |
| 3 | Self Disclosure/ or Help Seeking Behavior/ or (disclos* or nondisclos* or (non adj1 disclos*) or tell or tells or telling or told or reveal* or conceal* or hide or hides or hiding or hidden or secret* or secrecy or confid* or admit* or communicat* or (open* adj1 up) or ((help or support) adj3 seek*) or helpseek*).ti,ab. | Not yet executed |
| 4 | Qualitative Research/ or Interviews/ or Focus Group/ or (qualitative or interview* or (focus adj1 group*) or phenomenolog* or ethnograph* or (grounded adj1 theory) or ((thematic or content or framework) adj1 analys*) or (interpretative adj1 phenomenological adj1 analys*) or narrative* or (lived adj1 experience*) or experience* or perception* or perspective* or (mixed adj1 method*) or (open adj1 ended) or (action adj1 research) or (case adj1 stud*)).ti,ab. | Not yet executed |
| 5 | 1 and 2 and 3 | Not yet executed |
| 6 | 1 and 2 and 4 | Not yet executed |
| 7 | 5 or 6 | Not yet executed |
| Limits | Publication coverage through 31 March 2026; no other limits. | Not yet available |

| **CINAHL** Interface: EBSCOhost \| Formal search execution date: not yet executed | | |
| --- | --- | --- |
| **Search** | **Query** | **Records retrieved** |
| S1 | (MH "Self-Injurious Behavior+") OR TI ("nonsuicidal self-injury" OR "non-suicidal self-injury" OR "nonsuicidal self injury" OR "non suicidal self injury" OR NSSI OR "self injur*" OR "self-injur*" OR "self harm*" OR "self-harm*" OR selfinjur* OR selfharm* OR "self mutilat*" OR "self-mutilat*" OR "self cut*" OR "self-cut*" OR "deliberate self harm" OR DSH) OR AB ("nonsuicidal self-injury" OR "non-suicidal self-injury" OR "nonsuicidal self injury" OR "non suicidal self injury" OR NSSI OR "self injur*" OR "self-injur*" OR "self harm*" OR "self-harm*" OR selfinjur* OR selfharm* OR "self mutilat*" OR "self-mutilat*" OR "self cut*" OR "self-cut*" OR "deliberate self harm" OR DSH) | Not yet executed |
| S2 | (MH "Adolescence+") OR (MH "Child+") OR (MH "Young Adult") OR TI (adolescen* OR teen* OR youth* OR juvenile* OR "young person*" OR "young people" OR "young adult*" OR schoolchild* OR student*) OR AB (adolescen* OR teen* OR youth* OR juvenile* OR "young person*" OR "young people" OR "young adult*" OR schoolchild* OR student*) | Not yet executed |
| S3 | (MH "Self Disclosure") OR (MH "Help Seeking Behavior") OR TI (disclos* OR nondisclos* OR "non-disclos*" OR tell OR tells OR telling OR told OR reveal* OR conceal* OR hide OR hides OR hiding OR hidden OR secret* OR secrecy OR confid* OR admit* OR communicat* OR "opening up" OR "open up" OR "help seek*" OR "help-seek*" OR helpseek* OR "seek* help" OR "support seek*" OR "support-seek*") OR AB (disclos* OR nondisclos* OR "non-disclos*" OR tell OR tells OR telling OR told OR reveal* OR conceal* OR hide OR hides OR hiding OR hidden OR secret* OR secrecy OR confid* OR admit* OR communicat* OR "opening up" OR "open up" OR "help seek*" OR "help-seek*" OR helpseek* OR "seek* help" OR "support seek*" OR "support-seek*") | Not yet executed |
| S4 | (MH "Qualitative Studies+") OR (MH "Interviews+") OR (MH "Focus Groups") OR TI (qualitative OR interview* OR "focus group*" OR phenomenolog* OR ethnograph* OR "grounded theory" OR "thematic analys*" OR "content analys*" OR "framework analys*" OR "interpretative phenomenological analys*" OR narrative* OR "lived experience*" OR experience* OR perception* OR perspective* OR "mixed method*" OR "mixed-method*" OR "open ended" OR "open-ended" OR "action research" OR "case stud*") OR AB (qualitative OR interview* OR "focus group*" OR phenomenolog* OR ethnograph* OR "grounded theory" OR "thematic analys*" OR "content analys*" OR "framework analys*" OR "interpretative phenomenological analys*" OR narrative* OR "lived experience*" OR experience* OR perception* OR perspective* OR "mixed method*" OR "mixed-method*" OR "open ended" OR "open-ended" OR "action research" OR "case stud*") | Not yet executed |
| S5 | S1 AND S2 AND S3 AND S4 | Not yet executed |
| S6 | S1 AND S2 AND S4 | Not yet executed |
| S7 | S5 OR S6 | Not yet executed |
| Limits | Publication coverage through 31 March 2026; no other limits. | Not yet available |

| **Scopus** Interface: Elsevier Scopus \| Formal search execution date: not yet executed | | |
| --- | --- | --- |
| **Search** | **Query** | **Records retrieved** |
| #1 | TITLE-ABS-KEY("nonsuicidal self-injury" OR "non-suicidal self-injury" OR "nonsuicidal self injury" OR "non suicidal self injury" OR NSSI OR "self injur*" OR "self-injur*" OR "self harm*" OR "self-harm*" OR selfinjur* OR selfharm* OR "self mutilat*" OR "self-mutilat*" OR "self cut*" OR "self-cut*" OR "deliberate self harm" OR DSH) | Not yet executed |
| #2 | TITLE-ABS-KEY(adolescen* OR teen* OR youth* OR juvenile* OR "young person*" OR "young people" OR "young adult*" OR schoolchild* OR student*) | Not yet executed |
| #3 | TITLE-ABS-KEY(disclos* OR nondisclos* OR "non-disclos*" OR tell OR tells OR telling OR told OR reveal* OR conceal* OR hide OR hides OR hiding OR hidden OR secret* OR secrecy OR confid* OR admit* OR communicat* OR "opening up" OR "open up" OR "help seek*" OR "help-seek*" OR helpseek* OR "seek* help" OR "support seek*" OR "support-seek*") | Not yet executed |
| #4 | TITLE-ABS-KEY(qualitative OR interview* OR "focus group*" OR phenomenolog* OR ethnograph* OR "grounded theory" OR "thematic analys*" OR "content analys*" OR "framework analys*" OR "interpretative phenomenological analys*" OR narrative* OR "lived experience*" OR experience* OR perception* OR perspective* OR "mixed method*" OR "mixed-method*" OR "open ended" OR "open-ended" OR "action research" OR "case stud*") | Not yet executed |
| 5 | 1 AND 2 AND 3 | Not yet executed |
| 6 | 1 AND 2 AND 4 | Not yet executed |
| 7 | 5 OR 6 | Not yet executed |
| Limits | Publication coverage through 31 March 2026; no other limits. | Not yet available |

| **Web of Science Core Collection** Interface: Clarivate Web of Science \| Formal search execution date: not yet executed | | |
| --- | --- | --- |
| **Search** | **Query** | **Records retrieved** |
| #1 | TS=("nonsuicidal self-injury" OR "non-suicidal self-injury" OR "nonsuicidal self injury" OR "non suicidal self injury" OR NSSI OR "self injur*" OR "self-injur*" OR "self harm*" OR "self-harm*" OR selfinjur* OR selfharm* OR "self mutilat*" OR "self-mutilat*" OR "self cut*" OR "self-cut*" OR "deliberate self harm" OR DSH) | Not yet executed |
| #2 | TS=(adolescen* OR teen* OR youth* OR juvenile* OR "young person*" OR "young people" OR "young adult*" OR schoolchild* OR student*) | Not yet executed |
| #3 | TS=(disclos* OR nondisclos* OR "non-disclos*" OR tell OR tells OR telling OR told OR reveal* OR conceal* OR hide OR hides OR hiding OR hidden OR secret* OR secrecy OR confid* OR admit* OR communicat* OR "opening up" OR "open up" OR "help seek*" OR "help-seek*" OR helpseek* OR "seek* help" OR "support seek*" OR "support-seek*") | Not yet executed |
| #4 | TS=(qualitative OR interview* OR "focus group*" OR phenomenolog* OR ethnograph* OR "grounded theory" OR "thematic analys*" OR "content analys*" OR "framework analys*" OR "interpretative phenomenological analys*" OR narrative* OR "lived experience*" OR experience* OR perception* OR perspective* OR "mixed method*" OR "mixed-method*" OR "open ended" OR "open-ended" OR "action research" OR "case stud*") | Not yet executed |
| 5 | 1 AND 2 AND 3 | Not yet executed |
| 6 | 1 AND 2 AND 4 | Not yet executed |
| 7 | 5 OR 6 | Not yet executed |
| Limits | Publication coverage through 31 March 2026; no other limits. TS searches Topic fields. | Not yet available |

| **ProQuest Dissertations and Theses Global** Interface: ProQuest \| Formal search execution date: not yet executed | | |
| --- | --- | --- |
| **Search** | **Query** | **Records retrieved** |
| #1 | NOFT("nonsuicidal self-injury" OR "non-suicidal self-injury" OR "nonsuicidal self injury" OR "non suicidal self injury" OR NSSI OR "self injur*" OR "self-injur*" OR "self harm*" OR "self-harm*" OR selfinjur* OR selfharm* OR "self mutilat*" OR "self-mutilat*" OR "self cut*" OR "self-cut*" OR "deliberate self harm" OR DSH) | Not yet executed |
| #2 | NOFT(adolescen* OR teen* OR youth* OR juvenile* OR "young person*" OR "young people" OR "young adult*" OR schoolchild* OR student*) | Not yet executed |
| #3 | NOFT(disclos* OR nondisclos* OR "non-disclos*" OR tell OR tells OR telling OR told OR reveal* OR conceal* OR hide OR hides OR hiding OR hidden OR secret* OR secrecy OR confid* OR admit* OR communicat* OR "opening up" OR "open up" OR "help seek*" OR "help-seek*" OR helpseek* OR "seek* help" OR "support seek*" OR "support-seek*") | Not yet executed |
| #4 | NOFT(qualitative OR interview* OR "focus group*" OR phenomenolog* OR ethnograph* OR "grounded theory" OR "thematic analys*" OR "content analys*" OR "framework analys*" OR "interpretative phenomenological analys*" OR narrative* OR "lived experience*" OR experience* OR perception* OR perspective* OR "mixed method*" OR "mixed-method*" OR "open ended" OR "open-ended" OR "action research" OR "case stud*") | Not yet executed |
| 5 | 1 AND 2 AND 3 | Not yet executed |
| 6 | 1 AND 2 AND 4 | Not yet executed |
| 7 | 5 OR 6 | Not yet executed |
| Limits | Database restricted to ProQuest Dissertations and Theses Global; publication coverage through 31 March 2026, with no lower date bound and no language or full-text-availability limit. | Not yet available |

| **China National Knowledge Infrastructure** Interface: CNKI web advanced search \| Formal search execution date: not yet executed | | |
| --- | --- | --- |
| **Search** | **Query** | **Records retrieved** |
| A | In the 主题 field, search each term and combine with OR: 非自杀性自伤；非自杀性自损；非自杀性自残；非自杀性自我伤害；自伤；自残；自损；自我伤害；故意自伤；NSSI；self-injury；self-harm；nonsuicidal self injury. | Not yet executed |
| P | In the 主题 field, search each term and combine with OR: 青少年；少年；青年；儿童；未成年人；青春期；中学生；初中生；高中生；大学生；adolescent；teen；youth；young people；young adult. | Not yet executed |
| B | In the 主题 field, search each term and combine with OR: 披露；表露；自我表露；自我披露；告知；告诉；透露；倾诉；坦白；交流；沟通；隐瞒；隐匿；掩饰；隐藏；保密；秘密；求助；寻求帮助；寻求支持；disclosure；disclosing；telling；revealing；concealment；help seeking；help-seeking. | Not yet executed |
| Q | In the 主题 field, search each term and combine with OR: 质性；定性；访谈；焦点小组；扎根理论；现象学；民族志；叙事；主题分析；内容分析；框架分析；生活体验；亲身经历；经验；感受；看法；混合方法；开放式；qualitative；interview；focus group；thematic analysis；mixed methods；lived experience. | Not yet executed |
| 5 | A AND P AND B | Not yet executed |
| 6 | A AND P AND Q | Not yet executed |
| 7 | 5 OR 6 | Not yet executed |
| Limits | Run China Academic Journals, China Doctoral Dissertations and China Masters Theses separately and retain collection-specific yields. Select literal term matching; turn off intelligent/synonym expansion where available. No truncation or proximity syntax is assumed; save the interface-generated expression or screenshot. Apply the publication cutoff of 31 March 2026 as specified above. | Not yet available |

| **Wanfang Data** Interface: Wanfang web advanced search \| Formal search execution date: not yet executed | | |
| --- | --- | --- |
| **Search** | **Query** | **Records retrieved** |
| A | In the 主题 field, search each term and combine with OR: 非自杀性自伤；非自杀性自损；非自杀性自残；非自杀性自我伤害；自伤；自残；自损；自我伤害；故意自伤；NSSI；self-injury；self-harm；nonsuicidal self injury. | Not yet executed |
| P | In the 主题 field, search each term and combine with OR: 青少年；少年；青年；儿童；未成年人；青春期；中学生；初中生；高中生；大学生；adolescent；teen；youth；young people；young adult. | Not yet executed |
| B | In the 主题 field, search each term and combine with OR: 披露；表露；自我表露；自我披露；告知；告诉；透露；倾诉；坦白；交流；沟通；隐瞒；隐匿；掩饰；隐藏；保密；秘密；求助；寻求帮助；寻求支持；disclosure；disclosing；telling；revealing；concealment；help seeking；help-seeking. | Not yet executed |
| Q | In the 主题 field, search each term and combine with OR: 质性；定性；访谈；焦点小组；扎根理论；现象学；民族志；叙事；主题分析；内容分析；框架分析；生活体验；亲身经历；经验；感受；看法；混合方法；开放式；qualitative；interview；focus group；thematic analysis；mixed methods；lived experience. | Not yet executed |
| 5 | A AND P AND B | Not yet executed |
| 6 | A AND P AND Q | Not yet executed |
| 7 | 5 OR 6 | Not yet executed |
| Limits | Run journals and dissertations separately and retain collection-specific yields. Select literal term matching; turn off intelligent/synonym expansion where available. No truncation or proximity syntax is assumed; save the interface-generated expression or screenshot. Apply the publication cutoff of 31 March 2026 as specified above. | Not yet available |

| **Chinese Biomedical Literature Database** Interface: SinoMed \| Formal search execution date: not yet executed | | |
| --- | --- | --- |
| **Search** | **Query** | **Records retrieved** |
| A | In the 常用字段 field, search each term and combine with OR: 非自杀性自伤；非自杀性自损；非自杀性自残；非自杀性自我伤害；自伤；自残；自损；自我伤害；故意自伤；NSSI；self-injury；self-harm；nonsuicidal self injury. | Not yet executed |
| P | In the 常用字段 field, search each term and combine with OR: 青少年；少年；青年；儿童；未成年人；青春期；中学生；初中生；高中生；大学生；adolescent；teen；youth；young people；young adult. | Not yet executed |
| B | In the 常用字段 field, search each term and combine with OR: 披露；表露；自我表露；自我披露；告知；告诉；透露；倾诉；坦白；交流；沟通；隐瞒；隐匿；掩饰；隐藏；保密；秘密；求助；寻求帮助；寻求支持；disclosure；disclosing；telling；revealing；concealment；help seeking；help-seeking. | Not yet executed |
| Q | In the 常用字段 field, search each term and combine with OR: 质性；定性；访谈；焦点小组；扎根理论；现象学；民族志；叙事；主题分析；内容分析；框架分析；生活体验；亲身经历；经验；感受；看法；混合方法；开放式；qualitative；interview；focus group；thematic analysis；mixed methods；lived experience. | Not yet executed |
| 5 | A AND P AND B | Not yet executed |
| 6 | A AND P AND Q | Not yet executed |
| 7 | 5 OR 6 | Not yet executed |
| Limits | Search the Chinese Biomedical Literature Database only. Select literal term matching; turn off intelligent/synonym expansion where available. No truncation or proximity syntax is assumed; save the interface-generated expression or screenshot. Apply the publication cutoff of 31 March 2026 as specified above. | Not yet available |

| **Other information sources and update searches** Prospective methods \| Execution dates: not yet executed | | |
| --- | --- | --- |
| **Search** | **Query** | **Records retrieved** |
| Backward | Examine every reference in each included report and the three reviews cited in the Introduction (references 12, 14 and 17). Record each seed citation, date checked, references screened and records exported. | Not yet executed |
| Forward | Use Scopus Cited by for each included report and the three seed reviews. Record each seed citation/DOI, date, citing records screened and records exported; repeat for newly included studies. | Not yet executed |
| Repositories | Search the institutional repository associated with any potentially eligible thesis or study using its native all-metadata field. Run: "self-injury" disclosure; "self-harm" disclosure; NSSI qualitative; "self-injury" "help seeking"; "self-harm" concealment; 自伤 披露. Record repository name/URL, date, field, query, display limit, records screened and exported. | Not yet executed |
| Contacts | Contact corresponding authors of included studies and investigators identified from the three seed reviews to ask about completed or unpublished qualitative studies. Request report citations or access information only, not raw interviews, datasets or IPD. Record recipient role, date, request, reply and candidate reports. | Not yet executed |
| Updates | Rerun every database strategy before synthesis to identify newly indexed records published through 31 March 2026. Record every execution date, accepted history and yield. Extending the publication window requires a dated protocol amendment. | Not yet executed |

| **Deduplication and record management** Software: EndNote 20 and JBI SUMARI \| Formal records not yet available | | |
| --- | --- | --- |
| **Search** | **Query** | **Records retrieved** |
| 1 | Preserve every raw export with database, collection, route and run identifiers before merging in EndNote 20 (Clarivate). | Counts pending |
| 2 | Match exact DOI, PMID or other stable identifiers where present. | Counts pending |
| 3 | Run EndNote Find Duplicates using Author, Year, Title and Reference Type; retain the most complete record and preserve all source tags. | Counts pending |
| 4 | Manually verify remaining candidates using normalized title, first author, year, journal and pages, including Chinese-English variants. Remove only verified duplicate citations. | Counts pending |
| 5 | Link multiple reports from the same study rather than deleting them as duplicate citations. Archive the retained ID, removed ID, reason, reviewer and date in the duplicate log. | Counts pending |
| 6 | Report records retrieved and exported by source, total raw records, duplicate citations removed, unique records transferred to JBI SUMARI and any transfer discrepancy. | Counts pending |

| **Source execution log** Formal source dates and yields are intentionally unpopulated until execution | | |
| --- | --- | --- |
| **Search** | **Query** | **Records retrieved** |
| Preliminary PubMed term-development search | 31 March 2026; 438 records reported; preliminary only; exported count not recorded. | 438 reported |
| Formal PubMed | Execution date, exact source coverage, strategy version, retrieved/exported counts and export filename will be entered after execution. | Not yet executed |
| Embase/Ovid | Execution date, exact source coverage, strategy version, retrieved/exported counts and export filename will be entered after execution. | Not yet executed |
| APA PsycINFO/Ovid | Execution date, exact source coverage, strategy version, retrieved/exported counts and export filename will be entered after execution. | Not yet executed |
| CINAHL/EBSCOhost | Execution date, exact source coverage, strategy version, retrieved/exported counts and export filename will be entered after execution. | Not yet executed |
| Scopus | Execution date, exact source coverage, strategy version, retrieved/exported counts and export filename will be entered after execution. | Not yet executed |
| Web of Science Core Collection | Execution date, exact source coverage, strategy version, retrieved/exported counts and export filename will be entered after execution. | Not yet executed |
| ProQuest Dissertations and Theses Global | Execution date, exact source coverage, strategy version, retrieved/exported counts and export filename will be entered after execution. | Not yet executed |
| CNKI journals | Execution date, exact source coverage, strategy version, retrieved/exported counts and export filename will be entered after execution. | Not yet executed |
| CNKI doctoral dissertations | Execution date, exact source coverage, strategy version, retrieved/exported counts and export filename will be entered after execution. | Not yet executed |
| CNKI masters theses | Execution date, exact source coverage, strategy version, retrieved/exported counts and export filename will be entered after execution. | Not yet executed |
| Wanfang journals | Execution date, exact source coverage, strategy version, retrieved/exported counts and export filename will be entered after execution. | Not yet executed |
| Wanfang dissertations | Execution date, exact source coverage, strategy version, retrieved/exported counts and export filename will be entered after execution. | Not yet executed |
| CBM/SinoMed | Execution date, exact source coverage, strategy version, retrieved/exported counts and export filename will be entered after execution. | Not yet executed |
| Backward citation searching | Execution date, exact source coverage, strategy version, retrieved/exported counts and export filename will be entered after execution. | Not yet executed |
| Forward citation searching | Execution date, exact source coverage, strategy version, retrieved/exported counts and export filename will be entered after execution. | Not yet executed |
| Each named repository | Execution date, exact source coverage, strategy version, retrieved/exported counts and export filename will be entered after execution. | Not yet executed |
| Author/expert contacts | Execution date, exact source coverage, strategy version, retrieved/exported counts and export filename will be entered after execution. | Not yet executed |
| Search update runs | Execution date, exact source coverage, strategy version, retrieved/exported counts and export filename will be entered after execution. | Not yet executed |

| **PRISMA-S prospective reporting record** PRISMA-S guides the reporting of the planned and completed searches | | |
| --- | --- | --- |
| **Search** | **Query** | **Records retrieved** |
| 1-2 | Name each database and platform; report any multi-database searching. | Specified above; searches will be run one database/collection at a time. |
| 4-7 | Report online resources, citation searching, contacts and other methods. | Prospective methods are specified above; names, dates and yields will be completed after execution. |
| 8 | Provide every full strategy exactly as run. | Planned strategies are provided above; the final accepted histories will replace them verbatim after live execution. |
| 9-10 | Report publication-date limits, other restrictions, and any search filters. | Publication coverage through 31 March 2026; no other database limits. The complementary qualitative route is bespoke, not a validated filter. |
| 12-13 | Report update methods and the date of each search. | Every run and update will be dated in the source execution log. |
| 14 | Describe search peer review. | The search strategy has not undergone peer review using the Peer Review of Electronic Search Strategies (PRESS) framework. |
| 15 | Report records identified from every source. | The source execution log will be completed with retrieved and exported counts after execution. |
| 16 | Describe software and process used for deduplication. | EndNote 20, manual verification, source tagging and the duplicate audit log are specified above. |
| 3 | State whether study registries will be searched. | Study registry searches are not planned. |
| 11 | Identify searches adapted from prior work. | The limited preliminary PubMed search informed term development. The formal strategies are those specified in this appendix; no external published search filter is adopted. |

References. Rethlefsen ML, Kirtley S, Waffenschmidt S, et al. PRISMA-S: an extension to the PRISMA Statement for Reporting Literature Searches in Systematic Reviews. Syst Rev. 2021;10:39. doi:10.1186/s13643-020-01542-z. McGowan J, Sampson M, Salzwedel DM, Cogo E, Foerster V, Lefebvre C. PRESS Peer Review of Electronic Search Strategies: 2015 guideline statement. J Clin Epidemiol. 2016;75:40-46. doi:10.1016/j.jclinepi.2016.01.021.

# **Appendix II**

## JBI qualitative appraisal and dependability

Two reviewers will independently complete the JBI Critical Appraisal Checklist for Qualitative Research. Responses are Yes, No, Unclear, or Not applicable, with a supporting quotation or page locator. Study exclusion will not be based solely on appraisal scores. Disagreements will be resolved by discussion or a third reviewer.

| **Item** | **Appraisal question** | **ConQual item** |
| --- | --- | --- |
| Q1 | Is there congruity between the stated philosophical perspective and the research methodology? | No |
| Q2 | Is there congruity between the research methodology and the research question or objectives? | Yes |
| Q3 | Is there congruity between the research methodology and the methods used to collect data? | Yes |
| Q4 | Is there congruity between the research methodology and the representation and analysis of data? | Yes |
| Q5 | Is there congruity between the research methodology and the interpretation of results? | No |
| Q6 | Is there a statement locating the researcher culturally or theoretically? | Yes |
| Q7 | Is the influence of the researcher on the research, and vice-versa, addressed? | Yes |
| Q8 | Are participants, and their voices, adequately represented? | No |
| Q9 | Is the research ethical according to current criteria or, for recent studies, is there evidence of ethical approval by an appropriate body? | No |
| Q10 | Do the conclusions drawn in the research report flow from the analysis, or interpretation, of the data? | No |

Appraisal record per study and item: study ID, report and page, reviewer 1 judgment and reason, reviewer 2 judgment and reason, consensus judgment, and adjudication if required. Only Yes counts as a met dependability criterion. No, Unclear, and Not applicable do not count as met; reasons for Not applicable will be recorded without changing the five-item denominator. The ConQual rules are specified in Appendix IV.

Source: JBI Critical Appraisal Checklist for Qualitative Research and the JBI Manual for Evidence Synthesis (2024), cited in the manuscript.

# **Appendix III**

## Study characteristics and extraction records

Each study will have a stable study ID, with linked report IDs. Keep original findings, illustrations, contextual notes, and later DPM mappings separate. The primary outcome comprises synthesized findings on disclosure experiences, barriers, and facilitators; no additional outcomes are prespecified. These domains do not prescribe extraction categories.

| **Record** | **Fields to collect** |
| --- | --- |
| Study characteristics | Study/report IDs; citation; country and setting; methodology; data-collection and analysis methods; recruitment; sample size; age range and separately eligible adolescent subgroup; disclosure recipients and context. |
| NSSI eligibility | Primary terminology and definition; evidence of intentional direct tissue injury, absent suicidal intent, and exclusion of socially sanctioned injury; assessment or account establishing intent; source/page; eligible subgroup or behavior; separability decision; author clarification and final decision. |
| Disclosure eligibility | Episode/pathway ID; intentional and uncoerced communication; initiator; communication mode; recipient/audience; setting; prior observation or involuntary discovery; research-context disclosure; source and eligibility rationale. |
| Finding and illustration | Finding ID; verbatim author-reported analytic finding; verbatim supporting quotation, observation, or other illustration; participant/subgroup and episode attribution; page/table locator; context; reviewer credibility judgments and final rating. |
| Synthesis contribution | Final category ID and label; synthesized-finding ID; reason for grouping; source-to-category links; divergent or unaggregated status; dependability and credibility contributions. |
| Later DPM interpretation | Synthesized-finding ID; mapping status and proposed concept; rationale; contrasting or extending evidence; unresolved or unmapped status; mapping date; reviewer and adjudication record. |

Use study reports and public supplements; retain original and translated findings and illustrations with source locators. Record author clarifications separately, including the question, response, date, locator and decision affected. Do not obtain raw interviews, complete study datasets or IPD, or reanalyze quotations or clarifications as new primary findings. Summarize study characteristics and synthesized outcomes; retain extraction records in JBI SUMARI.

# Appendix IV

## ConQual decision rules and Summary of Findings

ConQual will be applied to each synthesized finding after meta-aggregation and before DPM mapping. Each starts at High confidence. Dependability and credibility downgrades are added, with a minimum final rating of Very low. Unsupported findings do not enter categorization, synthesis, or ConQual.

## Dependability

Use Q2, Q3, Q4, Q6, and Q7 in Appendix II. These assess congruity of methodology with the question, data collection, and data representation/analysis; the researcher’s cultural or theoretical positioning; and reciprocal researcher influence. Count only Yes as met.

| **Yes responses out of five** | **Study dependability** | **Downgrade** |
| --- | --- | --- |
| 4 or 5 | High | 0 |
| 2 or 3 | Moderate | 1 |
| 0 or 1 | Low | 2 |

Each eligible primary finding inherits its source-study dependability rating. For each synthesized finding, count each distinct contributing finding once, even if it has several illustrations or appears in linked reports. Apply the rating held by more than half of those findings. If no rating exceeds half, apply the lowest dependability rating represented. This tie/no-majority rule is prespecified for this review. Record both the finding counts and the number of distinct contributing studies at each level; do not use participant sample size as a weight.

## Credibility

Unequivocal findings have illustrations that clearly support the reported interpretation. Credible findings have an identifiable but contestable link between the interpretation and illustration. Unsupported findings lack adequate supporting data or illustration. Two reviewers will assess that link independently.

| **Composition of contributing findings** | **Credibility downgrade** |
| --- | --- |
| All Unequivocal | 0 |
| At least one Unequivocal and at least one Credible | 1 |
| All Credible | 2 |
| Unsupported | Excluded before synthesis; not a ConQual contributor |

## Final confidence and adjudication

A total downgrade of zero, one, two, or at least three levels gives High, Moderate, Low, or Very low confidence, respectively.

Two reviewers will independently record appraisal judgments, credibility ratings, contributor lists, both downgrades, and final ratings before comparison. Disagreements will be discussed against source reports and illustrations. A third reviewer will independently examine unresolved items and document the adjudication. Any change to an eligible contributor list will trigger reassessment of both ConQual domains; DPM mapping will not alter confidence ratings.

## Planned Summary of Findings fields

| **Field** | **Required content** |
| --- | --- |
| Review scope | Review title; adolescents aged 10-19 years with at least one voluntary NSSI disclosure; voluntary disclosure experiences, barriers and facilitators; all eligible contexts. |
| Synthesized finding | Finding ID and statement; linked categories; contributing primary-finding IDs and study/report IDs; research type (qualitative). |
| Dependability | Counts of findings and studies at each rating; majority or no-majority rule applied; downgrade and reason. |
| Credibility | Counts of Unequivocal and Credible findings; downgrade and reason. Unsupported findings are absent from these counts. |
| Final rating | High, Moderate, Low, or Very low; total downgrade; reviewer decisions and adjudication reference. |

Unsupported-finding log: study/report ID, finding ID, verbatim finding, available illustration or its absence, source locator, both reviewer judgments, final reason for exclusion, and any clarification received. Eligibility exclusions are recorded separately from Unsupported credibility ratings.

Source: Munn Z, Porritt K, Lockwood C, Aromataris E, Pearson A. Establishing confidence in the output of qualitative research synthesis: the ConQual approach. BMC Med Res Methodol. 2014;14:108. doi:10.1186/1471-2288-14-108.

# Appendix V

## NSSI and voluntary disclosure eligibility

Apply the NSSI criteria to the behavior being disclosed and the disclosure criteria to the communication episode. At study level, inclusion requires at least one separately extractable eligible finding with a supporting illustration. A study’s terminology alone does not determine eligibility.

## Acceptable NSSI definitions and evidence

NSSI is intentional, direct damage to one’s own body tissue without suicidal intent and outside socially sanctioned practices. An explicit compatible study definition or recruitment/assessment rule is sufficient when it applies to the findings being extracted and is not contradicted by the report. For studies using self-harm, deliberate self-harm, self-injury, self-injurious behavior, or self-injurious thoughts and behaviors, an assessment, clearly attributed account, or author clarification must establish that the relevant behavior satisfies this definition. A diagnostic NSSI-disorder threshold or a named instrument is not required.

The absence of suicidal intent must be stated or established for the eligible behavior or defined subgroup. It will not be inferred from the label alone, cutting as a method, low injury severity, an emotion-regulation function, or silence about suicide. Thoughts without an enacted NSSI behavior and self-poisoning without direct tissue injury do not meet the definition. A participant’s history of suicidal ideation or attempts does not exclude a clearly distinguishable NSSI disclosure. An act involving both suicidal and non-suicidal intent, or unresolved ambivalence about dying, is ineligible.

## Separability threshold

A mixed study is eligible only to the extent that an author-reported analytic finding and its supporting illustration can both be attributed to eligible adolescents, NSSI behavior, and voluntary disclosure. Attribution can be provided by the report, a clearly defined subgroup, or author clarification linked to the published material. A quotation from one eligible adolescent does not convert an overarching mixed NSSI/suicidal theme into an NSSI-specific finding. Reviewers will not construct new subgroup themes by reanalyzing individual quotations. No percentage of NSSI participants substitutes for separability.

Exclude inseparable mixed findings while retaining separable eligible findings from the same study. If no eligible findings remain, exclude the study and record the full-text reason. Apply eligibility before assessing credibility; uncertainty about intent is not resolved by assigning a lower credibility rating.

## Voluntary disclosure definition and taxonomy

Voluntary NSSI disclosure is an adolescent’s intentional communication of information about their own NSSI to a person, group, or online audience, either spontaneously or in response to a prompt, without coercion or compelled acknowledgment. Voluntariness refers to the adolescent’s decision to communicate, not to who initiated the interaction. Communication may be direct or indirect, verbal or nonverbal, and may occur face-to-face, digitally, or anonymously.

| **Episode category** | **Include when** | **Exclude or classify separately** | **Coding note** |
| --- | --- | --- | --- |
| Spontaneous disclosure | The adolescent raises or communicates information about their own NSSI without a preceding question or observation. | NSSI inferred from injuries or behavior without an intentional communication. | Record recipient or audience, setting, and stated purpose when available. |
| Prompted voluntary disclosure | The adolescent intentionally responds to a question, invitation, or prompt about their own NSSI. | A disclosure made under threat, force, punishment, or compelled acknowledgment. | Code as voluntary when the response is intentional and not coerced, even when another person initiated the conversation. |
| Observation-triggered disclosure | After scars or injuries are observed, the adolescent intentionally confirms, explains, or elaborates on their NSSI. | The observation itself, or an admission made only because the adolescent is compelled to respond. | Treat the observation as involuntary discovery and the later intentional communication as a separate episode when possible. |
| Indirect or nonverbal disclosure | The adolescent intentionally communicates through a note, drawing, gesture, deliberate display, or other nonverbal act, with communicative intent supported by the report. | An ambiguous signal interpreted as disclosure by researchers without evidence of the adolescent’s intent. | Extract the mode of communication and the evidence used to establish intent. |
| Digital or anonymous disclosure | The adolescent intentionally communicates through a direct message, online forum, helpline, social-media post, chat, or anonymous platform. | General online discussion of NSSI that is not attributable to the adolescent’s own experience. | Record whether the recipient or audience was identifiable, pseudonymous, or anonymous. |
| Research-context disclosure | The adolescent intentionally reports their own NSSI for the first time during a research interview or other qualitative data-collection interaction, and the study examines that report as a disclosure experience. | NSSI mentioned only in a screening item, demographic form, or participant characteristic with no disclosure account. | Include in the review when eligible, but code separately from disclosures in interpersonal, educational, clinical, or community settings. |
| Subsequent disclosure after involuntary discovery | After an earlier accidental observation, third-party report, or compelled acknowledgment, the adolescent later communicates their NSSI intentionally. | A later episode that cannot be distinguished from the original discovery or compelled response. | Include only the later intentional episode when it is separately identifiable. Record the earlier discovery as context. |
| Involuntary discovery | Information about NSSI becomes known through accidental observation, third-party reporting, or compelled acknowledgment. | Not eligible as voluntary disclosure. | Exclude from the voluntary disclosure synthesis unless a subsequent intentional episode is separately reported. |

## Integrated eligibility decision tree

| **Step** | **Question and decision** |
| --- | --- |
| 1 | Is there primary qualitative evidence attributable to adolescents aged 10-19 years? If no, exclude; if unclear, seek clarification at full text. |
| 2 | Does the disclosed behavior meet the NSSI definition, with affirmative evidence of absent suicidal intent? If yes, continue. If suicidal/mixed intent is explicit, exclude the affected evidence. If unclear, follow the contact procedure; unresolved evidence is excluded. |
| 3 | Did the adolescent intentionally communicate about their own NSSI, spontaneously or after a prompt, without coercion or compelled acknowledgment? If no, exclude that episode. |
| 4 | Is the communication direct, indirect, verbal, nonverbal, digital, or anonymous? Include any mode with supported communicative intent. For research-context disclosure, the study must examine the interaction as a disclosure experience; code it separately. |
| 5 | Was there earlier involuntary discovery? Include only a later intentional disclosure that is distinguishable from that discovery; retain the discovery as context. |
| 6 | Are the primary finding and supporting illustration separately attributable to eligible NSSI disclosure? If yes, retain for credibility appraisal. If inseparable or unresolved after clarification, exclude the finding. |
| 7 | Does the study retain at least one eligible finding with supporting illustration? If yes, include the study and assess credibility. If no, exclude with a recorded reason. Only Unequivocal and Credible findings proceed to meta-aggregation. |

## Inclusion and exclusion examples

| **Example** | **Decision** | **Reason** |
| --- | --- | --- |
| Asked by a school nurse, the adolescent explains that the scars resulted from NSSI. | Include | Prompted voluntary disclosure, if the response was intentional and not coerced. |
| A parent notices scars, and the adolescent intentionally confirms and explains the NSSI. | Include | Observation-triggered voluntary disclosure. Code the observation as preceding involuntary discovery. |
| A friend reports the adolescent’s NSSI to a counselor, and the adolescent is required to admit it. | Exclude | Third-party reporting and compelled acknowledgment are involuntary. |
| The adolescent later chooses to discuss the NSSI in a separate interview with a clinician. | Include | Subsequent voluntary disclosure, provided it is separately identifiable. |
| The adolescent posts anonymously in an online forum that they have been self-injuring and asks for support. | Include | Digital or anonymous disclosure of the adolescent’s own NSSI. |
| During a qualitative interview, the adolescent describes NSSI for the first time and discusses the decision to tell the interviewer. | Include | Research-context disclosure. Code separately from real-world recipient disclosures. |
| The participant selects NSSI on a screening questionnaire but provides no account of communicating it to anyone. | Exclude | Screening information alone is not a disclosure episode. |
| A study reports barriers among participants who disclosed and participants who never disclosed, but does not distinguish their quotations or findings. | Clarify | Seek author clarification. Exclude from synthesis if the findings and supporting illustrations remain inseparable by disclosure status. |

The following examples address NSSI intent and separability.

| **Reported situation** | **Decision and reason** |
| --- | --- |
| A study uses self-harm but explicitly defines all target episodes as intentional direct tissue injury without intent to die. | Include eligible disclosure findings if the definition applies to those findings and other eligibility criteria are met. |
| An adolescent with a previous suicide attempt describes a distinct episode of cutting without intent to die and its voluntary disclosure. | Include the separable NSSI disclosure; the suicide history remains contextual information. |
| A broad self-harm theme combines suicide attempts and NSSI, with one NSSI quotation but no separate analytic finding. | Exclude the mixed theme unless clarification establishes a separately attributable finding and illustration. |
| A report describes cutting to relieve distress but does not address intent to die. | Seek clarification; exclude the affected finding if intent remains unresolved. |
| A disclosed act involved both a wish to die and a wish to regulate emotion. | Exclude the act as mixed intent, even if other acts by that participant qualify as NSSI. |
| A report concerns suicidal or self-injury thoughts without a disclosed NSSI behavior. | Exclude; thoughts alone do not meet the target phenomenon. |

## Mixed episodes and extraction

Classify episodes and findings rather than assigning one label to an entire participant or study. A history of involuntary discovery, suicidal behavior, or non-disclosure does not invalidate a separately identifiable eligible voluntary NSSI disclosure. For mixed reports, extract only qualifying findings and illustrations. Record the communication initiator, mode, recipient/audience, setting, observed injuries, prior discovery, research context, and the NSSI definition and intent evidence. Keep unresolved eligibility exclusions outside the synthesis and the ConQual contributor lists.

## Author contact and unresolved cases

At full-text assessment or extraction, one reviewer will email the corresponding author with a citation, source locator, and a specific question about the definition, methods, intent, attribution, separability, or reported supporting material. Requests are limited to clarification of already reported evidence. Do not request or obtain raw interviews, complete study datasets, or IPD from investigators or repositories. Publicly available reports and supplements remain eligible sources. Clarifications are linked to the report and are not reanalyzed as new primary findings. Send one reminder on day 14 if no adequate reply has arrived. On day 28, two reviewers will decide using the available evidence. Record the contact address, dates, question, response, locator, and decision; a third reviewer will adjudicate unresolved disagreement.

Non-response is not itself an exclusion criterion when the report establishes eligibility. Unresolved intent or attribution excludes the affected finding; a study with no qualifying findings is excluded. Missing methodological detail is rated Unclear in appraisal. Missing support is handled by the credibility rules, including Unsupported when no adequate illustration is available. Apply the same two-contact, four-week schedule to methodological and extraction queries, combining questions for the same study where possible. Late clarifications received before the synthesis is finalized will be logged and reassessed by both reviewers.

# Appendix VI

## Synthesis sequence and screening calibration

## PICo review question

How do adolescents aged 10-19 years who have voluntarily disclosed NSSI describe their disclosure experiences, barriers, and facilitators across interpersonal, service, and digital contexts?

| **Element** | **Specification** |
| --- | --- |
| Population | Adolescents aged 10-19 years with a history of NSSI and at least one voluntary disclosure. |
| Phenomenon of interest | Lived experiences of voluntary NSSI disclosure and barriers and facilitators reported in relation to that disclosure. |
| Context | Interpersonal, family, peer, educational, healthcare, community, and digital settings in any geographic or sociocultural context. |

## Preserving findings before theoretical mapping

| **Stage** | **Procedure and record** |
| --- | --- |
| 1. Eligibility and extraction | Apply Appendix V. Retain the author-reported finding and its supporting illustration verbatim, with unique finding, report and study IDs. Separate original wording, translation, and contextual annotations. Do not assign DPM codes. |
| 2. Credibility appraisal | Independently rate each finding as Unequivocal, Credible, or Unsupported. Resolve disagreements. Remove Unsupported findings before categorization and keep the exclusion log. |
| 3. Categories | Group eligible Unequivocal/Credible findings by similarity of meaning and relevant context. Preserve source links and document grouping decisions. Do not use DPM components as preset headings. |
| 4. Synthesized findings | Aggregate categories into practical statements grounded in the primary interpretations. Retain differences by recipient, setting or disclosure pathway when combining would change meaning. Preserve divergent evidence. |
| 5. ConQual | Record contributor lists, domain downgrades and final confidence according to Appendix IV. Preserve a dated version of the synthesis and confidence judgments before DPM mapping. |
| 6. DPM interpretation | Compare the finalized synthesized findings with antecedent goals, disclosure events, outcomes and feedback processes. Record each proposed link and supporting rationale in a separate matrix. Mark partial, multiple, divergent or absent mappings explicitly. |

Two reviewers will conduct categorization and synthesis independently, compare their proposed groupings, and resolve differences against the original findings. A third reviewer will verify the evidence links and adjudicate unresolved disagreements. Prior familiarity with the DPM will be addressed in reflexive notes. DPM concepts will be used as sensitizing concepts only during the later interpretation stage.

Findings that contradict a category will prompt examination of its wording, context, and boundaries. Retain a separate category or report an eligible finding outside the aggregation when combination would erase the difference. Findings outside the DPM remain part of the review and may identify limits to the model’s applicability. Mapping is an interpretive comparison, not a test or validation of the model. It cannot change source wording, category membership, synthesized statements, or ConQual ratings. A factual extraction error discovered later must be corrected transparently, with affected synthesis and confidence judgments reassessed before mapping resumes.

## Screening pilot sample and decisions

Let N be the total number of deduplicated records available for title/abstract screening. Select a computer-generated random sample of 10% of N, rounded up, bounded by 100 and 200 records. If N is below 100, use all records. Record the sampling method, seed and selected record IDs. Each human reviewer independently assigns retain (potentially eligible or unclear) or exclude, records the reason, and locks decisions before comparison. A missing judgment must be completed before calculating agreement; it is not coded as exclusion.

Two human reviewers will make screening, extraction, appraisal, and confidence judgments independently. No machine model will substitute for either reviewer. Screening will not use machine-learning prioritization, eligibility recommendations, or automatic inclusion or exclusion. EndNote duplicate matching, random pilot sampling, and JBI SUMARI record management support this workflow; machine translation may support reading. Review judgments remain with the reviewers.

The bounds provide enough records to expose recurrent interpretive differences while limiting repeated training work in a large retrieval. These are prespecified operational calibration choices, not a power calculation or a claim that agreement establishes screening accuracy.

| **Reviewer 1 / Reviewer 2** | **Retain** | **Exclude** |
| --- | --- | --- |
| Retain | a: both retain | b: reviewer 1 retains only |
| Exclude | c: reviewer 2 retains only | d: both exclude |

Report n = a + b + c + d, the four cell counts, agreements a + d, disagreements b + c, and their percentages. Raw agreement is (a + d)/n. For binary nominal decisions, let p = (2a + b + c)/(2n), the pooled retain proportion. Gwet’s AC1 is [raw agreement - 2p(1-p)]/[1 - 2p(1-p)]. Use the analytic variance estimator described by Gwet (2008) for the 95% confidence interval, and record the calculation software and version when performed. AC1 is prespecified because exclusion-dominated screening can produce unstable kappa values despite high agreement. Also report retain-specific agreement 2a/(2a+b+c) and exclude-specific agreement 2d/(2d+b+c); a zero denominator is reported as not estimable.

## Passing and repeating calibration

A round passes when raw agreement is at least 80%, AC1 is at least 0.80, and every disagreement has been discussed with any necessary clarification documented. Thresholds apply to the locked pre-discussion decisions, not the consensus table. They are the review’s training criteria, not universal cutoffs. Report the confidence interval as uncertainty around AC1; it is not an additional passing threshold.

If either numerical criterion fails, identify whether disagreements concern age, NSSI intent, disclosure voluntariness, qualitative design, separability, or insufficient information. Clarify the screening instructions without altering review scope, then repeat independent calibration using a fresh random sample of the same target size. Use unused records first; if too few remain, include all unused records and randomly select previously used records to reach the target, documenting their number. For N below 100, re-randomize and reassess the full set after retraining. If there are no retrieved records, calibration is not applicable.

After three consecutive failed rounds, a third reviewer will examine the decision tables and lead further training. Calibration will continue until the criteria are met; adjudication will not substitute for passing a pilot. Retain all rounds, coefficients, intervals, disagreement reasons, guidance versions, and sampling records. Resolve pilot-record decisions under the final guidance before carrying them into formal screening. Borderline cases from Appendix V will be discussed in training separately from the random pilot and its agreement estimates.

Source: Gwet KL. Computing inter-rater reliability and its variance in the presence of high agreement. Br J Math Stat Psychol. 2008;61:29-48. doi:10.1348/000711006X126600.
